# Risk factors for inguinal hernia repair among US adults

**DOI:** 10.1007/s10029-023-02913-w

**Published:** 2023-11-10

**Authors:** B. Cowan, M. Kvale, J. Yin, S. Patel, E. Jorgenson, R. Mostaedi, H. Choquet

**Affiliations:** 1 UCSF-East Bay General Surgery, Oakland, CA USA; 2 Institute for Human Genetics, University of California, San Francisco, San Francisco, CA USA; 3 Division of Research, Kaiser Permanente Northern California (KPNC), Oakland, CA USA; 4 Regeneron Genetics Center, Tarrytown, NY USA; 5 KPNC, Richmond Medical Center, Richmond, CA USA

**Keywords:** Inguinal hernia, Hernia repair, Epidemiology of inguinal hernia, Inguinal hernia risk factors

## Abstract

**Purpose:**

To investigate demographic, clinical, and behavioral risk factors for undergoing inguinal hernia repair within a large and ethnically diverse cohort.

**Methods:**

We conducted a retrospective case–control study from 2007 to 2020 on 302,532 US individuals from a large, integrated healthcare delivery system with electronic health records, who participated in a survey of determinants of health. Participants without diagnosis or procedure record of an inguinal hernia at enrollment were included. We then assessed whether demographic (age, sex, race/ethnicity), clinical, and behavioral factors (obesity status, alcohol use, cigarette smoking and physical activity) were predictors of undergoing inguinal hernia repair using survival analyses. Risk factors showing statistical significance (*P* < 0.05) in the univariate models were added to a multivariate model.

**Results:**

We identified 7314 patients who underwent inguinal hernia repair over the study period, with a higher incidence in men (6.31%) compared to women (0.53%). In a multivariate model, a higher incidence of inguinal hernia repair was associated with non-Hispanic white race/ethnicity, older age, male sex (aHR = 13.55 [95% confidence interval 12.70–14.50]), and more vigorous physical activity (aHR = 1.24 [0.045]), and alcohol drinker status (aHR = 1.05 [1.00–1.11]); while African-American (aHR = 0.69 [0.59–0.79]), Hispanic/Latino (aHR = 0.84 [0.75–0.91]), and Asian (aHR = 0.35 [0.31–0.39]) race/ethnicity, obesity (aHR = 0.33 [0.31–0.36]) and overweight (aHR = 0.71 [0.67–0.75]) were associated with a lower incidence. The use of cigarette was significantly associated with a higher incidence of inguinal hernia repair in women (aHR 1.23 [1.09–1.40]), but not in men (aHR 0.96 [0.91–1.02]).

**Conclusion:**

Inguinal hernia repair is positively associated with non-Hispanic white race/ethnicity, older age, male sex, increased physical activity, alcohol consumption and tobacco use (only in women); while negatively associated with obesity and overweight status. Findings from this large and ethnically diverse study may support future prediction tools to identify patients at high risk of this surgery.

**Supplementary Information:**

The online version contains supplementary material available at 10.1007/s10029-023-02913-w.

## Introduction

Inguinal hernia, which is defined as protrusion of abdominal contents through the muscle and fascial layers of the lower abdominal wall, is a common condition affecting all ages [1, 2]. Lifetime risk of development of groin hernias, which includes inguinal and femoral hernias, is 27% for men and 3% for women. Inguinal hernia repair is one of the most common operation performed by general surgeons, with more than one million repair operations taking place in the United States every year [3, 4].

Surgical inguinal hernia repair presents short-term and long-term risks to the patient. While watchful waiting can be an acceptable option for minimally symptomatic male patients, untreated inguinal hernias can lead to serious medical morbidity, including emergencies such as bowel incarceration or strangulation [1]. Inguinal hernia repair in the emergency setting carries substantially risk of mortality, twice as high as elective repair [4–6]. In regards to the long-term risk of inguinal hernia repair, a recent review study consisted of 25 studies (6293 participants) found that 2–4% of patients have hernia recurrence after repair (with and without mesh, respectively) and 5–10% of patients suffer from postoperative chronic pain [7, 8]. Investigating and understanding risk factors for inguinal hernia repair is a necessary step toward the prevention of morbidity relating to inguinal hernia.

Inguinal hernia development has a multifactorial etiology with interplay of environmental, genetic, and behavioral risk factors [2, 9, 10]. Epidemiologic risk factors for inguinal hernia have been previously demonstrated in only a few studies [2–4]. Several risk factors associated with development of inguinal hernia in adults have been identified, including older age, male sex, chronic obstructive pulmonary disease, lower body mass index (BMI), and family history [2, 4, 11–13]. Just one prior epidemiological study evaluated risk of inguinal hernia in relation to race, and only included breakdown of black and white ethnic groups [2].

Here we used data from a large, integrated healthcare delivery system, which incorporates health plan coverage with coordinated medical services, to examine the association of demographic, clinical, or behavioral risk factors, with inguinal hernia repair. The aim of this study was to investigate factors associated with inguinal hernia repair incidence and to provide a foundation for better surgical and preventative treatments.

## Methods

### Study population

Study sample was selected from the Kaiser Permanente Research Program on Genes, Environment, and Health (RPGEH), recruited from adult (at least 18 years-old) members of Kaiser Permanente Medical Care Plan, Northern California Region (KPNC). The RPGEH was established as a resource for research on genetic and environmental influences on health and disease, and all RPGEH participants completed a five-page mailed survey in 2007. All survey data, that gathered information on sociodemographic factors and health-related behaviors, have been linked to clinical data from electronic health records (EHR) of the participants. The sample in this study is a subset of the RPGEH identified as having no diagnosis or procedure records of inguinal hernia in KPNC at their enrollment in RPGEH. The participants provided their written informed consent to participate in this study. The Institutional Review Board of the Kaiser Foundation Research Institute has approved all study procedures (FWA# 00002344 IRB# 00001045).

### Risk factors

The covariates were collected from a survey taken by the participants at enrollment in RPGEH, or EHR, and included: age at survey (modelled as a categorical variable by dividing age into as the following categories 18- < 30; 30- < 40; 40- < 50; 50- < 60; 60- < 70; and 80 years old and plus), sex, race/ethnicity, BMI, alcohol consumption, cigarette smoking, and physical activity.

On the RPGEH survey, participants were asked: ‘What best describes your race/ethnicity?’ Self-reported race/ethnicity for each individual was derived from responses to this question, and, for individuals who reported more than one category, the selections were collapsed into race/ethnicity categories. In particular, all East Asian nationalities (i.e., Chinese, Japanese, Korean, Filipino, Vietnamese, or other Southeast Asian) were collapsed into a single East Asian group; all Latino nationalities (i.e., Mexican, Central/South American, Puerto Rican, or other Latino/Hispanic) were collapsed into a single Hispanic/Latino category; all African descent populations (i.e., African-American, African, or Africo-Caribbean) were collapsed into a single group; and all white-European ethnicities (i.e., White or European-American, Middle Eastern, or Ashkenazi Jewish) were collapsed into a single non-Hispanic white group [14].

Participants’ height and weight were recorded in the EHR and BMI was calculated as the weight in kilograms (kg) divided by the square of height in meters (m) [15]. Eligible patients were classified according to their BMI class, as follows: underweight for participants having a BMI < 20 kg/m^2^, normal weight for participants having a BMI ≥ 20 and < 25 kg/m^2^, overweight for participants having a BMI ≥ 25 and < 30 kg/m^2^, and obese for participants having a BMI ≥ 30 kg/m^2^.

Alcohol consumption (drinker/non-drinker status) was also assessed based on the RPGEH survey. On this survey, participants were asked regarding the past year: ‘On average, how many days a week do you have a drink containing alcohol?’ (no days, 1 day, 2 days, 3 days, 4 days, 5 days, 6 days or every day). Further, participants were asked: ‘On a typical day that you drink, how many drinks do you have?’ (none, 1, 2, 3, 4, 5, 6, 7 or ⩾8). Individuals who reported drinking at least 1 day per week and at least 1 drink per day were defined as ‘drinkers’, whereas those who provided negative answers (‘no days’ and ‘none’) were considered as ‘non-drinkers’ [10].

Smoking initiation was assessed based on the RPGEH survey, via the following questions: ‘Have you ever smoked one or more cigarettes per day for six months or longer?’ (yes or no); ‘Do you currently smoke, or have you stopped smoking?’ (current smoker or former smoker); the study population was divided into ever smokers (former/current) and never smokers [16].

Physical activity was assessed based on the RPGEH survey, via the following questions:

- During the past 7 days, on how many days did you walk or do exercise, sports or other physical activity that required moderate physical effort (other than walking), for at least 10 min at a time, fast enough to cause your heart rate to increase somewhat? (None or 1–2 or 3–4 or 5–6 or Every day).
- During the past 7 days, on how many days did you do exercise, sports or other physical activity for at least 10 min at a time that was vigorous enough to work up a sweat or cause your heart rate to increase substantially? (None or 1–2 or 3–4 or 5–6 or Every day).
- On average, how many minutes did you spend walking each day you walked / doing other moderate physical activity / doing vigorous physical activity each day you did it? (10–19 or 20–29 or 30–59 or 60 or more).
- During the past 7 days, how much time per day outside of work did you spend watching TV, videos or DVDs, using a computer, reading, driving, or riding in a car or other vehicle? (Less than 1 h or 3 h up to 5 h or 1 h up to 3 h or More than 5 h).

The total Metabolic Equivalent of Task (MET) in minutes per week was then estimated using the answers to the above-mentioned questions on physical activity and sedentariness and using a previously validated method [17]. Briefly, recreational physical activity summary scores were derived by multiplying assigned MET values by duration and frequency and summing across activities [17, 18]. We defined vigorous activities as participating in a minimum of 1260 MET-hours per week, on average, equivalent to at least 3.5 h of activity with a minimum MET level of 6. We defined moderate and vigorous activities combined as participating in a minimum of 630 MET-hours of activity per week, on average, equivalent to at least 3.5 h of activity with a minimum MET level of 3. In the current study, we assigned participants to 4 quartiles for MET (i.e. Q1 [1st quartile], Q2, Q3, and Q4), with Q4 corresponding to the physically more active group.

### Study outcome

The incidence of an inguinal hernia repair was the outcome measure, and participants were assigned as cases if they had an inguinal hernia repair during the study period; all non-cases were assigned to the control group. The study period (follow-up time) was calculated in years from the date when RPGEH survey was taken (2007) to the date of first inguinal hernia repair surgery for the cases; and to the last encounter date in KPNC for controls. The data was collected with a cutoff date for survival of October 5, 2020, totaling twelve years of data. Patients with inguinal hernia repair were identified in the KPNC electronic health record system based on the following International Classification of Disease (ICD) and Current Procedural Terminology (CPT) codes: at least one of the diagnosis codes for inguinal hernia coded as ICD9: 550 or ICD10: K40; and at least one of the procedure codes for open or laparoscopic repair of hernia ICD9: 53.0, 53.1, 17.1, 17.2 or CPT: 49491, 49492, 49495, 49496, 49500, 49501, 49505, 49507, 49520, 49521, 49525, 49650, 49651, 49659.

### Statistical analyses

The study dataset was prepared with SAS 9.4. Descriptive statistics were calculated for demographic and other risk factors for inguinal hernia repair incidence status. Univariate differences between inguinal hernia repair cases and controls were assessed with Pearson’s chi-squared χ^2^ tests. Factors showing statistical significance (*P* < 0.05) in the univariate models were added to a multivariate model in an effort to reduce bias from confounding variables. Cox proportional hazards regression models were created to assess the effect of each risk factor on the time to inguinal hernia repair procedure. Further, sex stratified analyses were conducted among women and men, separately. Survival curves were generated with the Kaplan–Meier method. All tests of statistical significance reported are 2-sided. All analyses were performed using the R software version 4.2.1 [19].

## Results

### Baseline patient characteristics

A total of 302,532 subjects were eligible for analysis, and, among those, 7314 (2.41%) underwent inguinal hernia repair during the 12.5 years follow-up. Patient characteristics are reported in Table 1. The mean age ± SD at survey completion for the study cohort was 58.4 years ± 16.1 and the incidence in men (6.3%) was almost twelve times higher than in women (0.53%). The majority of patients who underwent inguinal hernia repair were non-Hispanic whites (85%), followed by Hispanic/Latinos (6.6%;), East Asians (5.0%), and African Americans (2.7%). We also observed a higher incidence of inguinal hernia repair among non-Hispanic whites (2.8%) compared to Hispanic/Latinos (1.7%), African Americans (1.5%), and East Asians (1.0%).

**Table 1:** Characteristics of Kaiser Permanente RPGEH participants included in the study

	Controls n = 295,218	Cases n = 7314	Total n = 302,532	*P* Value
Age at survey, mean (SD)	58.2 (16.1)	64.7 (12.3)	58.4 (16.1)	< 0.001
Age at survey group, n (%)				
18- < 30	16,134 (5.5%)	44 (0.6%)	16,178 (5.3%)	
30- < 40	26,042 (8.8%)	193 (2.6%)	26,235 (8.7%)	
40- < 50	45,572 (15.4%)	719 (9.8%)	46,291 (15.3%)	
50- < 60	66,416 (22.5%)	1531 (20.9%)	67,947 (22.5%)	
60- < 70	66,343 (22.5%)	2127 (29.1%)	68,470 (22.6%)	
70- < 80	48,508 (16.4%)	2005 (27.4%)	50,513 (16.7%)	
80 +	26,203 (8.9%)	695 (9.5%)	26,898 (8.9%)	
Sex, n (%)				< 0.001
Female	202,621 (68.6%)	1077 (14.7%)	203,698 (67.3%)	
Male	92,597 (31.4%)	6237 (85.3%)	98,834 (32.7%)	
Race/ethnicity, n (%)				< 0.001
Non-Hispanic White	218,128 (73.9%)	6224 (85.1%)	224,352 (74.2%)	
East Asian	35,500 (12.0%)	364 (5.0%)	35,864 (11.9%)	
Hispanic/Latino	27,222 (9.2%)	481 (6.6%)	27,703 (9.2%)	
African American	12,853 (4.4%)	198 (2.7%)	13,051 (4.3%)	
Other	1515 (0.5%%)	47 (0.6%)	1562 (0.5%)	
BMI, n (%)				< 0.001
Underweight	5047 (1.7%)	67 (0.9%)	5114 (1.7%)	
Normal weight	117,929 (39.9%)	3046 (41.6%)	120,975 (40.0%)	
Overweight	100,569 (34.1%)	3336 (45.6%)	103,905 (34.3%)	
Obese	71,673 (24.3%)	865 (11.8%)	72,538 (24.0%)	
Alcohol Consumption, n (%)				
Non-drinker	146,041 (49.5%)	2640 (36.1%)	148,681 (49.1%)	
Drinker	149,177 (50.5%)	4674 (63.9%)	153,851 (50.9%)	
Smoking initiation				< 0.001
Ever smokers	111,467 (61.6%)	3300 (46.8%)	114,767 (38.6%)	
Never smokers	178,689 (38.4%)	3747 (53.2%)	182,436 (61.4%)	
Total MET, n (%)				< 0.001
1st Quartile	74,324 (25.2%)	1309 (17.9%)	75,633 (25.0%)	
2nd Quartile	73,955 (25.1%)	1678 (22.9%)	75,633 (25.0%)	
3rd Quartile	73,638 (24.9%)	1995 (27.3%)	75,633 (25.0%)	
4th Quartile	73,301 (24.8%)	2332 (31.9%)	75,633 (25.0%)	

Cases are those who underwent inguinal hernia repair surgery

SD standard deviation, MET metabolic equivalent of task

### Univariate models

We assessed whether age at survey completion (age groups), sex, race/ethnicity, BMI categories, alcohol use, cigarette smoking and physical activity were associated with inguinal hernia repair incidence. Univariate hazard ratios and 95% confidence intervals using Cox regression models showed that age group 70- < 80 (HR: 14.7, 95%CI: 10.9–19.8), male gender (HR: 12.2, 95%CI: 12.4–14.1), the use of alcohol (HR: 1.65, 95%CI 1.6–1.7) and cigarettes (HR: 1.5, 95%CI: 1.4–1.6), and more vigorous physical activity (total MET in the fourth quartile of the cohort) (HR: 1.60, 95%CI: 1.5–1.7), were independently associated with increased inguinal hernia repair incidence (Table 2). Survival curves for each risk factor are reported in the Supplementary Information (Supplementary Figures S1-S7). Further, non-Hispanic whites had an increased inguinal hernia repair incidence, compared to African-Americans, Hispanic/Latinos, and Asians. Obesity was associated with a lower incidence of inguinal hernia repair (HR: 0.47, 95%CI: 0.43–0.50).

**Table 2:** Risk factors associated with inguinal hernia repair incidence in the Kaiser Permanente RPGEH sample

Factors	Univariate model		Multivariate model	
	Hazard ratio (95%CI)	*P* value	Hazard ratio (95%CI)	*P* value
Age at survey group				
18- < 30	Reference		Reference	
30- < 40	2.44 (1.76–3.39)	< 0.001	2.53 (1.82–3.52)	< 0.001
40- < 50	5.00 (3.68–6.76)	< 0.001	4.42 (3.24–6.01)	< 0.001
50- < 60	7.31 (5.42–9.87)	< 0.001	6.95 (5.13–9.42)	< 0.001
60- < 70	10.32 (7.66–13.91)	< 0.001	9.44 (6.97–12.78)	< 0.001
70- < 80	14.70 (10.90–19.82)	< 0.001	11.96 (8.83–16.20)	< 0.001
80 +	13.33 (9.83–18.09)	< 0.001	9.94 (7.29–13.55)	< 0.001
Sex				
Female	Reference		Reference	
Male	13.21 (12.39–14.10)	< 0.001	13.57 (12.70–14.50)	< 0.001
Race/ethnicity				
Non-Hispanic White	Reference		Reference	
Asian	0.34 (0.31–0.38)	< 0.001	0.35 (0.31–0.39)	< 0.001
Hispanic/Latino	0.60 (0.55–0.66)	< 0.001	0.83 (0.75–0.91)	< 0.001
African American	0.53 (0.46–0.61)	< 0.001	0.69 (0.59–0.79)	< 0.001
Other	1.12 (0.84–1.49)	0.45	1.02 (0.76–1.37)	0.94
BMI				
Normal weight	Reference		Reference	
Underweight	0.59 (0.46–0.75)	< 0.001	0.79 (0.61–1.01)	0.06
Overweight	1.27 (1.21–1.33)	< 0.001	0.71 (0.67–0.75)	< 0.001
Obese	0.47 (0.43–0.50)	< 0.001	0.33 (0.31–0.36)	< 0.001
Alcohol consumption				
Non-drinker	Reference		Reference	
drinker	1.65 (1.58–1.73)	< 0.001	1.05 (1.00–1.11)	0.046
Smoking initiation				
Never smokers	Reference		Reference	
Ever smokers	1.50 (1.43–1.57)	< 0.001	0.99 (0.94–1.04)	0.46
Total MET				
Q1	Reference		Reference	
Q2	1.21 (1.13–1.30)	< 0.001	1.11 (1.03–1.20)	< 0.001
Q3	1.39 (1.30–1.49)	< 0.001	1.17 (1.09–1.26)	< 0.001
Q4	1.60 (1.55–1.71)	< 0.001	1.24 (1.15–1.33)	< 0.001

Multivariate model is adjusted for sex and race/ethnicity. BMI = body mass index. 95%CI = 95% confidence interval. MET = metabolic equivalent of task and Q1 refers to the first quartile of population stratified by MET (least active) and so on

CI confidence interval; MET metabolic equivalent of task; Q1, Q2, Q3, Q4 1st quartile, 2nd quartile, etc.

### Multivariate models and sex-specific analyses

**Fig. 1:**
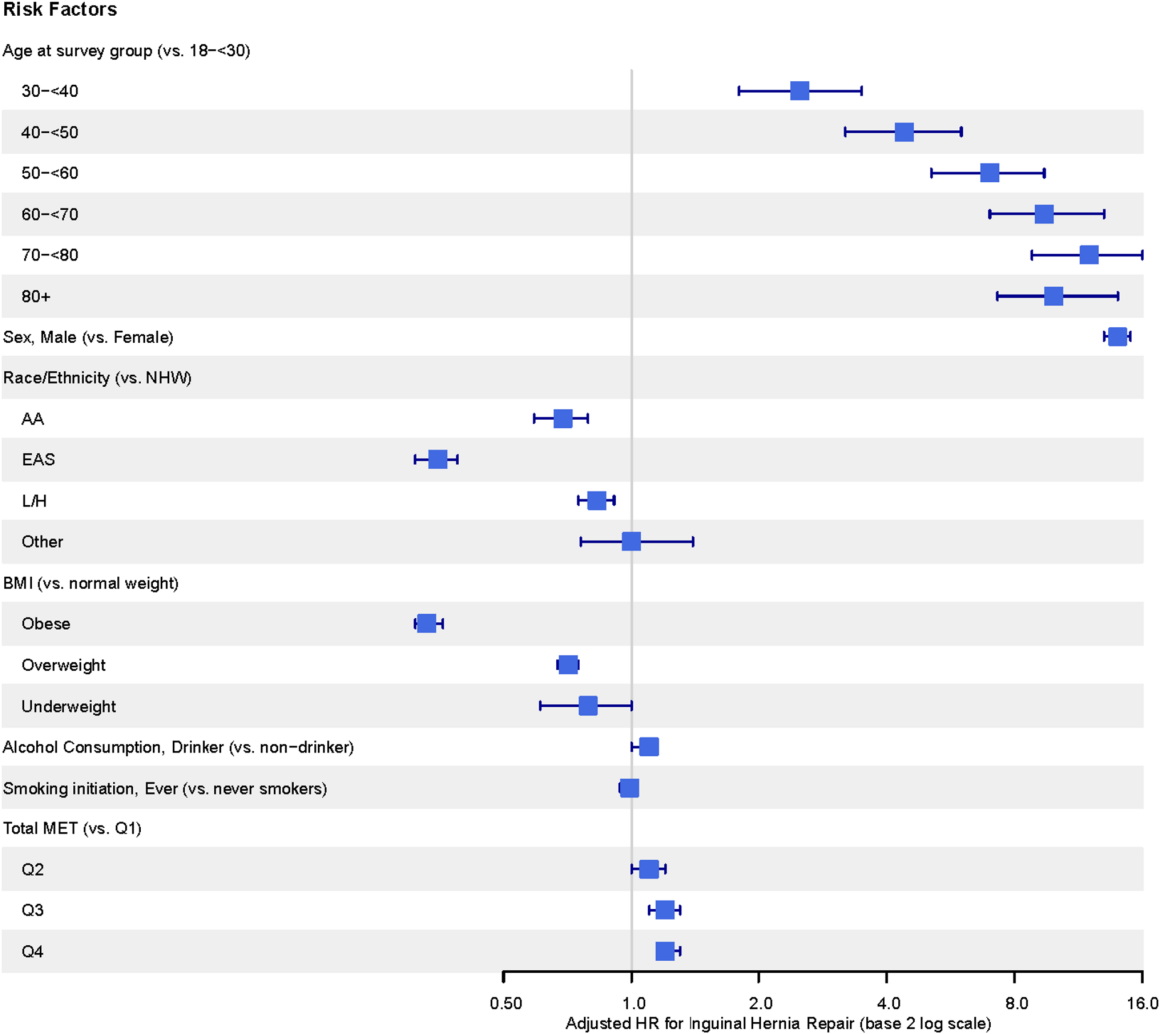
Hazard models of predictors of inguinal hernia repair according to multivariate model. HR hazard ratio; NHW non-Hispanic white; AA African American; EAS Asian; L/H Latin/Hispanic; MET metabolic equivalent of task; Q1, Q2, Q3, Q4 1st quartile, 2nd quartile, etc.

In a multivariate analysis, age group 70- < 80 (*P* < 0.001), male gender (*P* < 0.001), non-Hispanic white race/ethnicity group (*P* < 0.001), the use of alcohol (*P* = 0.046), and more vigorous physical activity (*P* < 0.001) remained significantly associated with a higher incidence of inguinal hernia repair, whereas obesity (*P* < 0.001) remained associated with a lower incidence of inguinal hernia repair (Table 2 and Fig. 1). In contrast, cigarette smoking was no longer a predictor of inguinal hernia repair incidence.

Given that men have an increased risk of inguinal hernia compared to women [1], we also conducted analyses stratified by sex. Similar results were observed between men and women in multivariate models, except for smoking initiation (Supplementary Tables S1-S2). The use of cigarette was significantly associated with a higher incidence of inguinal hernia repair in women (HR 1.23, *P* < 0.001), while this association was not observed in men (HR 0.96, *P* = 0.168) (Supplementary Figures S8-S9).

## Discussion

In this study of a large and ethnically diverse cohort of over 300,000 privately insured patients in the United States, 2.42% of patients underwent primary inguinal hernia repair over the study period, with a higher incidence in men (6.31%) compared to women (0.53%). In a multivariate analysis, older age, and specifically age group 70- < 80, male gender, non-Hispanic white race/ethnicity group, and more vigorous physical activity were associated with an increased incidence of inguinal hernia repair. In contrast, obesity was associated with a decreased incidence of inguinal hernia repair. Alcohol use was associated with a higher incidence of inguinal hernia repair overall, and tobacco use was associated with increased risk in women only.

Inguinal hernia repair in this study was noted to have a higher incidence in non-Hispanic whites with a statistically significant lower rate in all other race/ethnicities. To the best of our knowledge, our study is the first to quantify inguinal hernia repair risk among as varied of race/ethnic groups (i.e., non-Hispanic white, Hispanic/Latino, East Asian, and African American), as previous studies have included mostly non-Hispanic whites [2, 4, 5]. Although a higher rate of inguinal hernia repairs does not necessarily indicate a higher actual incidence of inguinal hernia occurrence, Ruhl and colleagues observed an association between an increased risk of inguinal hernia in whites compared to non-whites [2]. Future large and ethnically diverse studies will be needed to further confirm our findings.

Several of our results are consistent with previous studies. For one, inguinal hernia repair is much more common in men rather than women; the HR of 13 found in this study is even more pronounced than what is seen in the Ruhl study (HR 7.5), the Nilsson study (HR 10), the Primatesta study (HR 9) and the Zendejas study (HR 7) [2, 4–6]. Increasing rate of inguinal hernia repair as age increases corresponds to prior studies [2, 7]. In our study, rate of hernia repair increases with increasing age with a peak at age 70–79; this peak around age 70 was also demonstrated clearly in a large Danish study [20]. The reasons of the decrease in inguinal hernia repair incidence after age 80 is not identified in this study, but speculation includes surgeon discretion considering the increasing risk of anesthesia complications (up to 10%) in patients older than age 80 years [21].

Our multivariate analysis demonstrated an inverse relationship of BMI with rate of inguinal hernia repair. There was a statistically significant decrease in rate of inguinal hernia repair with increasing BMI. Normal weight individuals were the most likely to have inguinal hernia repair surgery. This effect has been shown in prior studies [2, 10]. A recent genetic investigation of inguinal hernia susceptibility demonstrates a shared genetic influence between BMI and inguinal hernia, and likely a causal effect of lower BMI and higher risk of inguinal hernia [9]. Some biological processes underlying BMI variation including impaired adipogenesis or insulin-signaling pathways may have consequences on inguinal hernia development. Additionally, we speculate that intraabdominal obesity may be a protective factor in inguinal hernia symptoms, protecting from incarceration of bowel. Regardless, the reason for this relationship could be the subject for further study.

Physical activity is one of the risk factors for inguinal hernia development with the most historical discussion. Flich in 1992 categorized various occupations with respect to the effort expended performing that job, and found that those in higher activity occupations were more likely to develop inguinal hernia [22]. Later studies did not find statistical significance in variation of hernia repair rate when associated with recreational effort, and varying results when considering non-recreational effort expended [2]. A recent genetic investigation of inguinal hernia susceptibility did find a shared genetic influence of inguinal hernia and increased physical activity level [9]. In our study, in a multivariate model, we found that increasing activity level as measured by MET is associated with increasing rate of inguinal hernia repair. MET in our study does not discriminate between leisure or work activity. This likely corresponds with historical results that suggest that increased activity leads to increased risk of inguinal hernia repair.

Our study reported, for the first time to our knowledge, sex-specific risk factors associated with inguinal hernia repair incidence. Largely, associations between inguinal hernia repair incidence and risk factors were similar between both sexes, except for tobacco use which was associated with increased risk of inguinal hernia repair only in women. Follow-up investigations in independent cohorts could confirm this sex-specific association and provide insights into the underlying mechanisms.

We recognize several potential limitations of our study. First, age at survey, sex, race/ethnicity, alcohol consumption, cigarette smoking, and physical activity risk factors were based on self-reported data, which may result in misclassification. Second, bias due to evaluation error could have arisen as alcohol consumption, cigarette smoking, and physical activity were assessed only once (at survey completion). Finally, as with all observational studies, associations do not indicate cause-and-effect relationships and could be related to unobserved population factors.

Nevertheless, our study is based on a unique and very large cohort of individuals, who were all members of the KPNC health plan, a single integrated healthcare delivery system. Participants were recruited in a similar manner and were evaluated for demographic, clinical, and behavior risk factors using a single questionnaire providing a great consistency.

Risk factors for inguinal hernia repair include white ethnicity, increasing age, alcohol use, tobacco use (only in women), and increasing physical activity. In contrast, having a higher BMI seems to have a protective effect. Identification of these risk factors can help to guide screening practices and may support future prediction tools to identify patients at high risk of this surgery. In addition, the identification of modifiable factors, such as alcohol and tobacco use could be promoted to reduce risk of inguinal hernia repair in addition to the multitude health issues compounded by these behaviors.

In conclusion, there are several established risk factors for inguinal hernia repair. Our study provides some insight and updates to this data. This is the first study to identify risk of inguinal hernia repair among more than two race/ethnic groups, which may improve generalizability among more populations. Finally, we hope our findings can help guide screening and counseling regarding this condition.

### Supplementary Information

Below is the link to the electronic supplementary material.

Supplementary file1 (DOCX 1147 KB)

## References

[1] Fitzgibbons RJ, Forse RA (2015). Groin hernias in adults. N Engl J Med.

[2] Ruhl CE, Everhart JE (2007). Risk factors for inguinal hernia among adults in the US population. Am J Epidemiol.

[3] Rutkow IM (2003). Demographic and socioeconomic aspects of hernia repair in the United States in 2003. Surg Clin N Am.

[4] Zendejas B, Ramirez T, Jones T (2013). Incidence of inguinal hernia repairs in Olmsted County, MN: a population-based study. Ann Surg.

[5] Primatesta P, Goldacre N (1997). Inguinal hernia repair: incidence of elective and emergency surgery, readmission and mortality. Int J Epidemiol.

[6] Nilsson H, Nilsson E, Angerås U, Nordin P (2011). Mortality after groin hernia surgery: delay of treatment and cause of death. Hernia.

[7] Lockhart K, Dunn D, Teo S (2018). Mesh versus non-mesh for inguinal and femoral hernia repair. Cochrane Database Syst Rev.

[8] Kehlet H, Jensen TS, Woolf CJ (2006). Persistent postsurgical pain: risk factors and prevention. Lancet.

[9] Jorgenson E, Thai KK, Hoffmann TJ (2017). Genetic contributors to variation in alcohol consumption vary by race/ethnicity in a large multi-ethnic genome-wide association study. Mol Psychiatry.

[10] Choquet H, Li W, Yin J (2022). Ancestry- and sex-specific effects underlying inguinal hernia susceptibility identified in a multiethnic genome-wide association study meta-analysis. Hum Mol Genet.

[11] Lau H, Fang C, Yuen WK, Patil NG (2007). Risk factors for inguinal hernia in adult males: a case-control study. Surgery.

[12] Rosemar A, Angerås U, Rosengren A (2008). Body mass index and groin hernia: a 34-year follow-up study in Swedish men. Ann Surg.

[13] Zöller B, Ji J, Sundquist J, Sundquist K (2013). Shared and nonshared familial susceptibility to surgically treated inguinal hernia, femoral hernia, incisional hernia, epigastric hernia, and umbilical hernia. J Am Coll Surg.

[14] Banda Y, Kvale MN, Hoffmann TJ (2015). Characterizing race/ethnicity and genetic ancestry for 100,000 subjects in the genetic epidemiology research on adult health and aging (GERA) cohort. Genetics.

[15] Hoffmann TJ, Choquet H, Yin J (2018). A large multiethnic genome-wide association study. Genetics.

[16] Choquet H, Yin J, Jorgenson E (2021). Cigarette smoking behaviors and the importance of ethnicity and genetic ancestry. Transl Psychiatry.

[17] Sidney S, Jacobs DR, Haskell WL (1991). Comparison of two methods of assessing physical activity in the Coronary Artery Risk Development in Young Adults (CARDIA) Study. Am J Epidemiol.

[18] Ainsworth BE, Haskell WL, Herrmann SD (2011). 2011 Compendium of Physical Activities: a second update of codes and MET values. Med Sci Sports Exerc.

[19] R Core Team (2022) R: A Language and Environment for Statistical. R Foundation for Statistical Computing, Vienna, Austria. https://www.R-project.org

[20] Burcharth J, Pedersen M, Bisgaard T, Pedersen C, Rosenberg J. Nationwide prevalence of groin hernia repair. PloS one. 2013 Jan 14;8(1):e54367. 10.1371/journal.pone.0054367 10.1371/journal.pone.0054367 PMC3544713 23342139

[21] Perez AJ, Campbell S (2022) Inguinal hernia repair in older persons. J Am Med Dir Assoc 23(4):563–567. 10.1016/j.jamda.2022.02.008 10.1016/j.jamda.2022.02.008 35259338

[22] Flich J, Alfonso JL, Delgado F, Prado MJ, Cortina P (1992) Inguinal hernia and certain risk factors. Eur J Epidemiol. 8(2):277-282. 10.1007/bf00144814 10.1007/BF00144814 1644149

