# Supplementary material for: Risk factors for inguinal hernia repair among US adults
Source: Hernia. 2023 Nov 10;27(6):1507–14. doi: 10.1007/s10029-023-02913-w (PMC10700424; doi:10.1007/s10029-023-02913-w)

**Supplementary Information**

**Cowan B. et al. Risk factors for inguinal hernia repair among US adults**

**Supplementary Table 1.** Risk factors associated with inguinal hernia repair incidence in Kaiser Permanente RPGEH women

**Supplementary Table 2.** Risk factors associated with inguinal hernia repair incidence in Kaiser Permanente RPGEH men

**Supplementary Fig. 1.** Kaplan–Meier inguinal hernia repair-specific survival probabilities by age

**Supplementary Fig. 2.** Kaplan–Meier inguinal hernia repair-specific survival probabilities by sex

**Supplementary Fig. 3.** Kaplan–Meier inguinal hernia repair-specific survival probabilities by race/ethnicity

**Supplementary Fig. 4.** Kaplan–Meier inguinal hernia repair-specific survival probabilities by BMI categories

**Supplementary Fig. 5.** Kaplan–Meier inguinal hernia repair-specific survival probabilities by alcohol use

**Supplementary Fig. 6.** Kaplan–Meier inguinal hernia repair-specific survival probabilities by cigarette smoking

**Supplementary Fig. 7.** Kaplan–Meier inguinal hernia repair-specific survival probabilities by physical activity

**Supplementary Fig. 8.** Hazard models of predictors of inguinal hernia repair (Women only)

**Supplementary Fig. 9.** Hazard models of predictors of inguinal hernia repair (Men only)

**Supplementary Table 1.** Risk factors associated with inguinal hernia repair incidence in Kaiser Permanente RPGEH women

|  | | Univariate models | | Multivariate model | |
| --- | --- | --- | --- | --- | --- |
| **Factors** | | **Hazard Ratio (95%CI)** | ***P* Value** | **Hazard Ratio (95%CI)** | ***P* Value** |
| Age at survey group | | Reference | | Reference | |
| 18-<30 | |  |  |  |  |
| 30-<40 | | 2.02 (0.97, 4.2) | 0.062 | 2.14 (1.02, 4.48) | 0.044 |
| 40-<50 | | 2.80 (1.40, 5.6) | **<0.001** | 2.88 (1.44, 5.75) | **<0.001** |
| 50-<60 | | 4.07 (2.08, 8.0) | **<0.001** | 4.13 (2.11, 8.10) | **<0.001** |
| 60-<70 | | 7.53 (3.88, 14.6) | **<0.001** | 7.34 (3.79, 14.35) | **<0.001** |
| 70-<80 | | 14.80 (7.64, 28.7) | **<0.001** | 14.17 (7.30, 27.52) | **<0.001** |
| 80+ | | 15.13 (7.71, 29.7) | **<0.001** | 14.07 (7.15, 27.71) | **<0.001** |
| Race/ethnicity | Non-Hispanic White | Reference | | Reference | |
|  | Asian | 0.20 (0.14, 0.28) | **<0.001** | 0.25 (0.17, 0.36) | **<0.001** |
|  | Hispanic/Latino | 0.78 (0.63, 0.96) | 0.021 | 1.14 (0.92, 1.42) | 0.231 |
|  | African American | 0.58 (0.41, 0.82) | **<0.001** | 0.81 (0.57, 1.16) | 0.250 |
|  | Other | 1.74 (0.93, 3.25) | 0.081 | 2.03 (1.09, 3.79) | 0.026 |
| BMI | Normal Weight | Reference | | Reference | |
|  | Underweight | 0.75 (0.47, 1.20) | 0.227 | 0.75 (0.47, 1.22) | 0.253 |
|  | Overweight | 0.80 (0.70, 0.91) | 0.001 | 0.69 (0.60, 0.79) | **<0.001** |
|  | Obese | 0.48 (0.40, 0.57) | **<0.001** | 0.45 (0.38, 0.54) | **<0.001** |
| Alcohol Consumption | Non-Drinker | Reference | | Reference | |
|  | Drinker | 1.25(1.1, 1.4) | **<0.001** | 1.05 (0.93, 1.19) | 0.435 |
| Smoking initiation | Never smokers | Reference | | Reference | |
|  | Ever smokers | 1.58 (1.4, 1.8) | **<0.001** | 1.23 (1.09, 1.40) | **<0.001** |
| Total MET | Q1 | Reference | | Reference | |
|  | Q2 | 1.20 (1.01, 1.4) | 0.038 | 1.21 (1.02, 1.45) | 0.029 |
|  | Q3 | 1.20 (1.01, 1.4) | 0.034 | 1.19 (1.00, 1.43) | 0.049 |
|  | Q4 | 1.18 (0.99, 1.4) | 0.058 | 1.22 (1.02, 1.47) | 0.030 |

Abbreviations: *CI* confidence interval; *MET* metabolic equivalent of task; *Q1, Q2, Q3, Q4* 1^st^ quartile, 2^nd^ quartile, etc.

**Supplementary Table 2.** Risk factors associated with inguinal hernia repair incidence in Kaiser Permanente RPGEH men

|  | | Univariate models | | Multivariate model | |
| --- | --- | --- | --- | --- | --- |
| **Factors** | | **Hazard Ratio (95%CI)** | ***P* Value** | **Hazard Ratio (95%CI)** | ***P* Value** |
| Age at survey group | |  |  |  |  |
| 18-<30 | | Reference | | Reference | |
| 30-<40 | | 2.36 (1.6, 3.4) | **<0.001** | 2.63 (1.82, 3.81) | **<0.001** |
| 40-<50 | | 4.30 (3.1, 6.1) | **<0.001** | 4.75 (3.36, 6.71) | **<0.001** |
| 50-<60 | | 6.78 (4.9, 9.5) | **<0.001** | 7.62 (5.43, 10.73) | **<0.001** |
| 60-<70 | | 9.01 (6.4, 12.6) | **<0.001** | 9.96 (7.09, 14.00) | **<0.001** |
| 70-<80 | | 11.04 (7.9, 15.4) | **<0.001** | 11.66 (8.29, 16.40) | **<0.001** |
| 80+ | | 9.62 (6.8, 13.5) | **<0.001** | 9.37 (6.62, 13.28) | **<0.001** |
| Race/ethnicity | Non-Hispanic White |  |  | Reference | |
|  | Asian | 0.359 (0.32, 0.40) | **<0.001** | 0.363 (0.32, 0.41) | **<0.001** |
|  | Hispanic/Latino | 0.610 (0.55, 0.68) | **<0.001** | 0.782 (0.70, 0.87) | **<0.001** |
|  | African American | 0.554 (0.47, 0.65) | **<0.001** | 0.663 (0.57, 0.78) | **<0.001** |
|  | Other | 0.897 (0.65, 1.24) | 0.511 | 0.893 (0.64, 1.25) | 0.513 |
| BMI | Normal Weight |  |  | Reference | |
|  | Underweight | 0.758 (0.57, 1.01) | 0.055 | 0.799 (0.60, 1.07) | 0.130 |
|  | Overweight | 0.787 (0.75, 0.83) | **<0.001** | 0.704 (0.67, 0.74) | **<0.001** |
|  | Obese | 0.341 (0.31, 0.37) | **<0.001** | 0.313 (0.29, 0.34) | **<0.001** |
| Alcohol Consumption | Non-Drinker |  |  | Reference | |
|  | Drinker | 1.23 (1.2, 1.3) | **<0.001** | 1.05 (1.00, 1.11) | 0.053 |
| Smoking initiation | Never smokers |  |  | Reference | |
|  | Ever smokers | 1.16 (1.1, 1.2) | **<0.001** | 0.963 (0.91, 1.02) | 0.168 |
| Total MET | Q1 |  |  | Reference | |
|  | Q2 | 1.13 (1.0, 1.2) | 0.002 | 1.10 (1.01, 1.19) | 0.022 |
|  | Q3 | 1.23 (1.1, 1.3) | **<0.001** | 1.17 (1.09 1.27) | **<0.001** |
|  | Q4 | 1.32 (1.2, 1.4) | **<0.001** | 1.24 (1.15, 1.34) | **<0.001** |

Abbreviations: *CI* confidence interval; *MET* metabolic equivalent of task; *Q1, Q2, Q3, Q4* 1^st^ quartile, 2^nd^ quartile, etc.

**Supplementary Fig. 1.** Kaplan–Meier inguinal hernia repair-specific survival probabilities by age


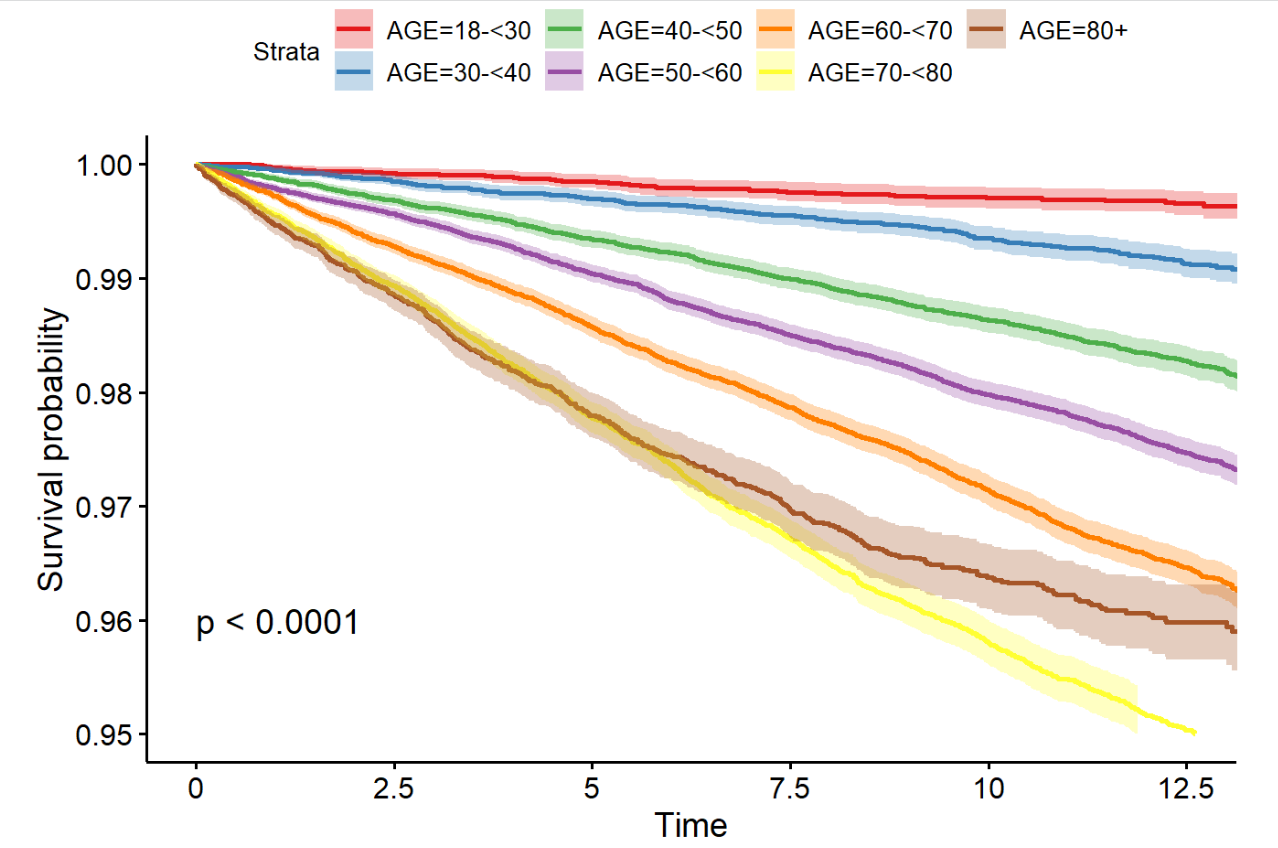


**Supplementary Fig. 2.** Kaplan–Meier inguinal hernia repair-specific survival probabilities by sex


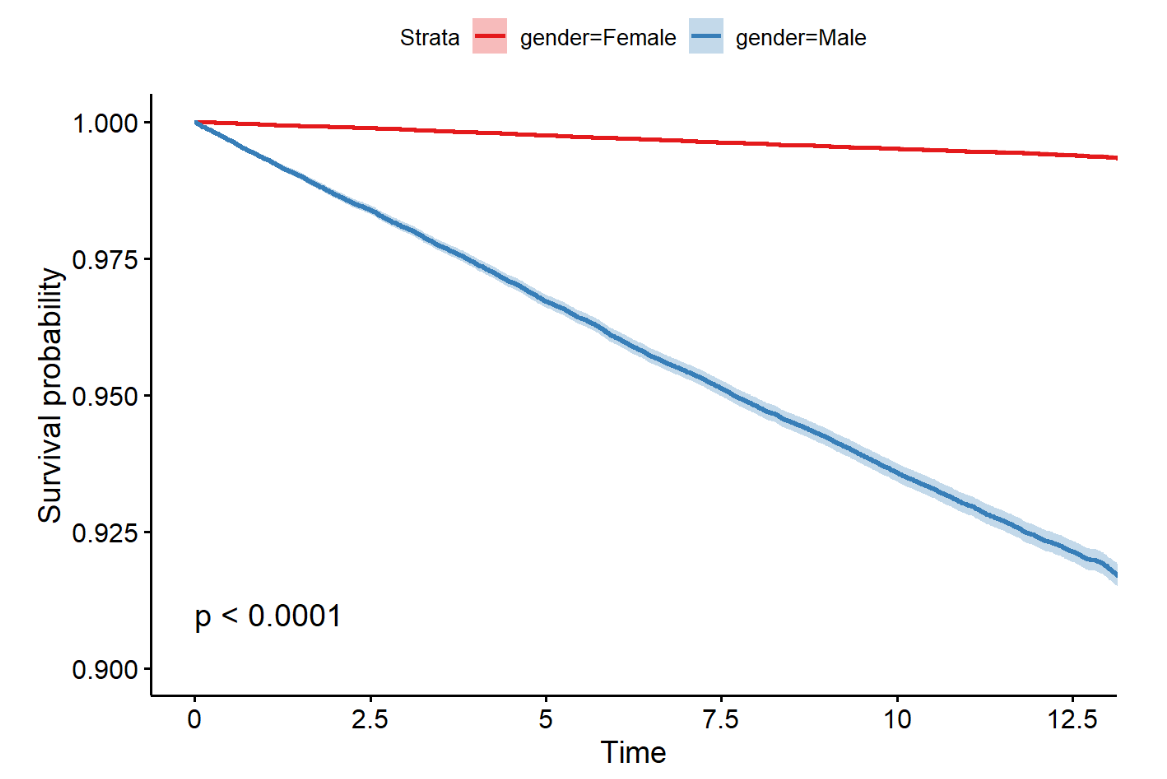


**Supplementary Fig. 3.** Kaplan–Meier inguinal hernia repair-specific survival probabilities by race/ethnicity


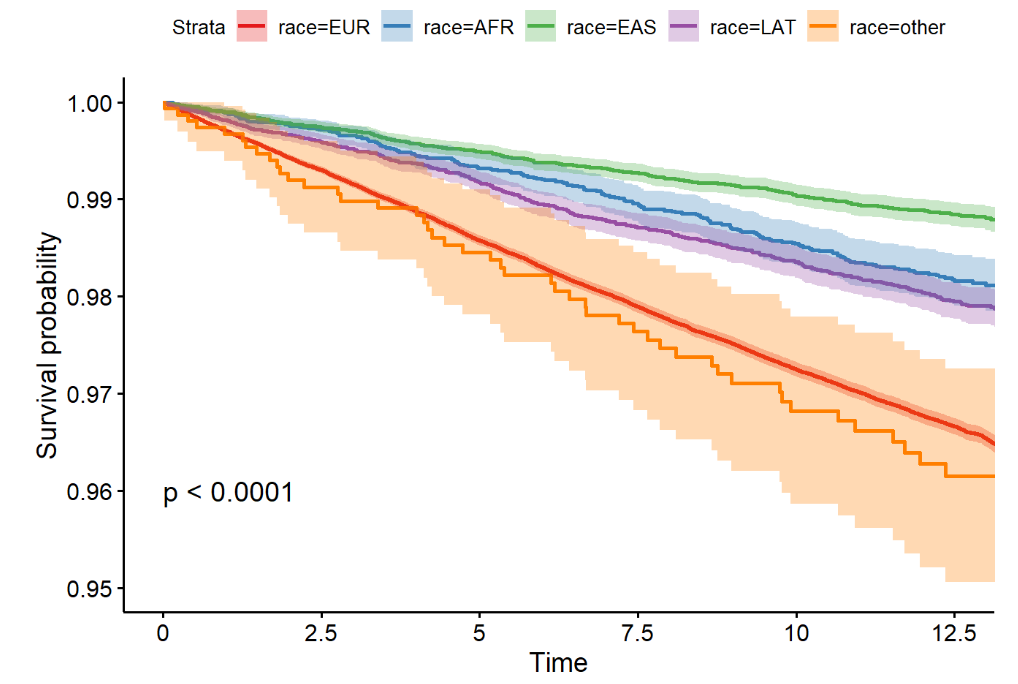


**Supplementary Fig. 4.** Kaplan–Meier inguinal hernia repair-specific survival probabilities by BMI categories


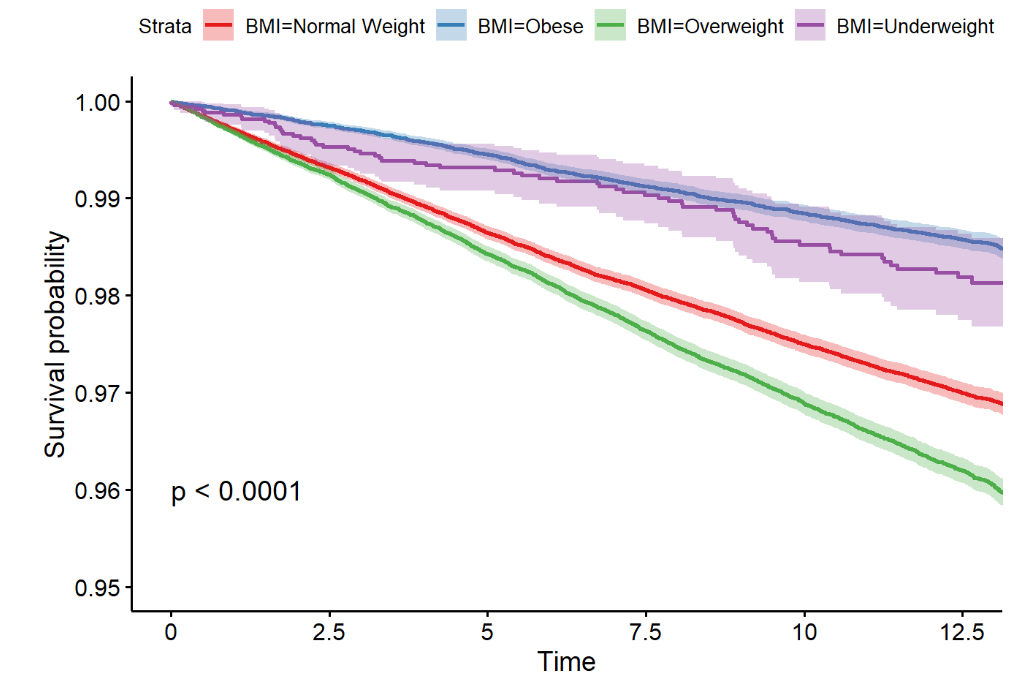


**Supplementary Fig. 5.** Kaplan–Meier inguinal hernia repair-specific survival probabilities by alcohol use


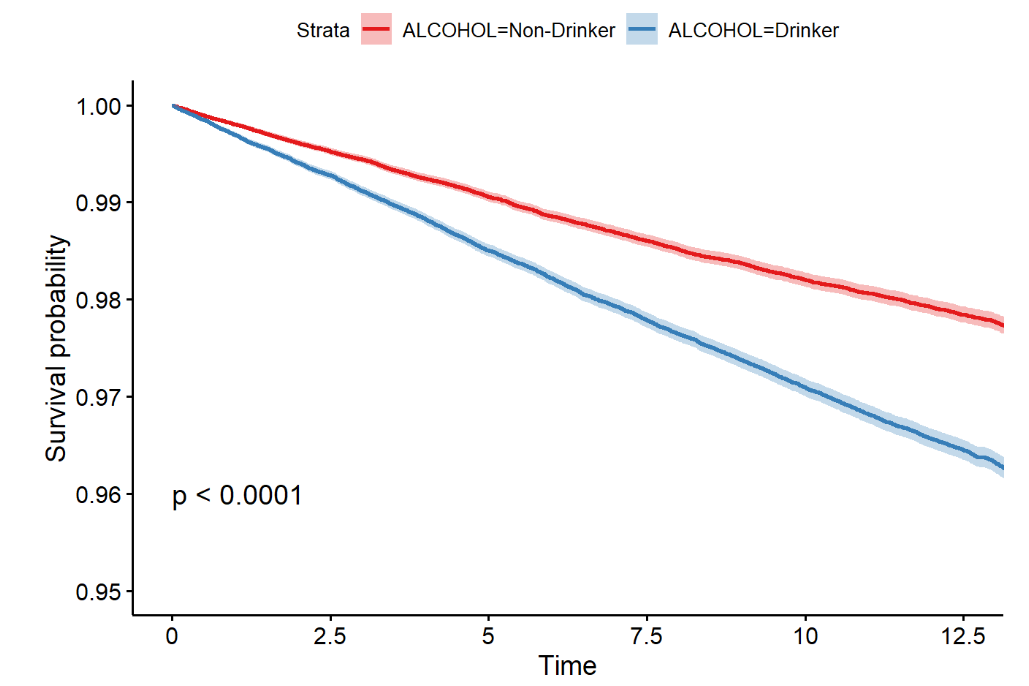


**Supplementary Fig. 6.** Kaplan–Meier inguinal hernia repair-specific survival probabilities by cigarette smoking


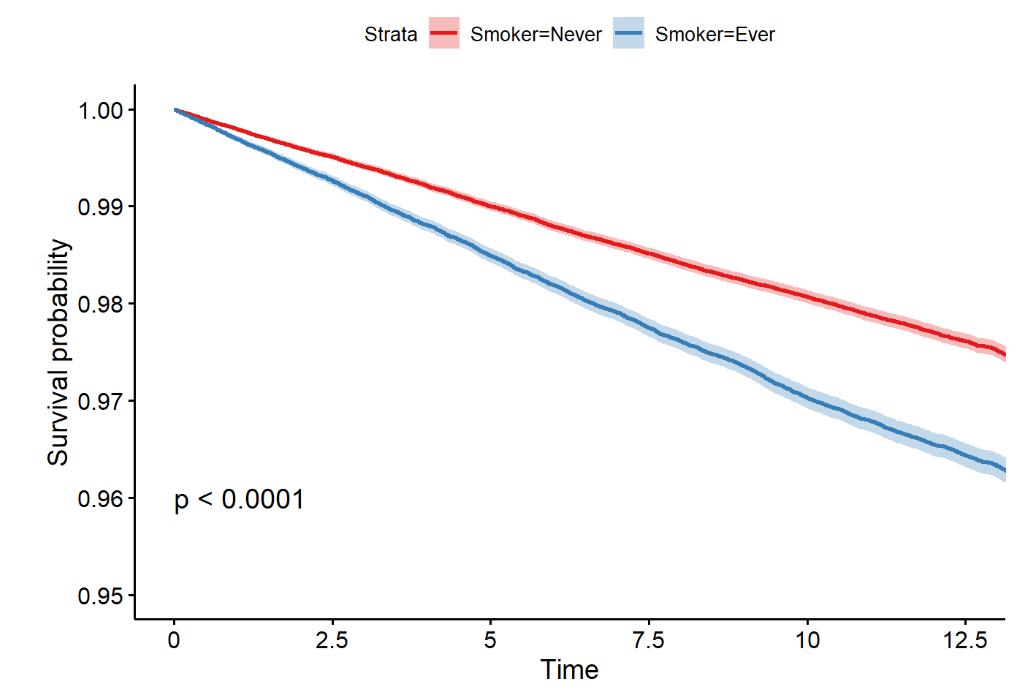


**Supplementary Fig. 7.** Kaplan–Meier inguinal hernia repair-specific survival probabilities by physical activity


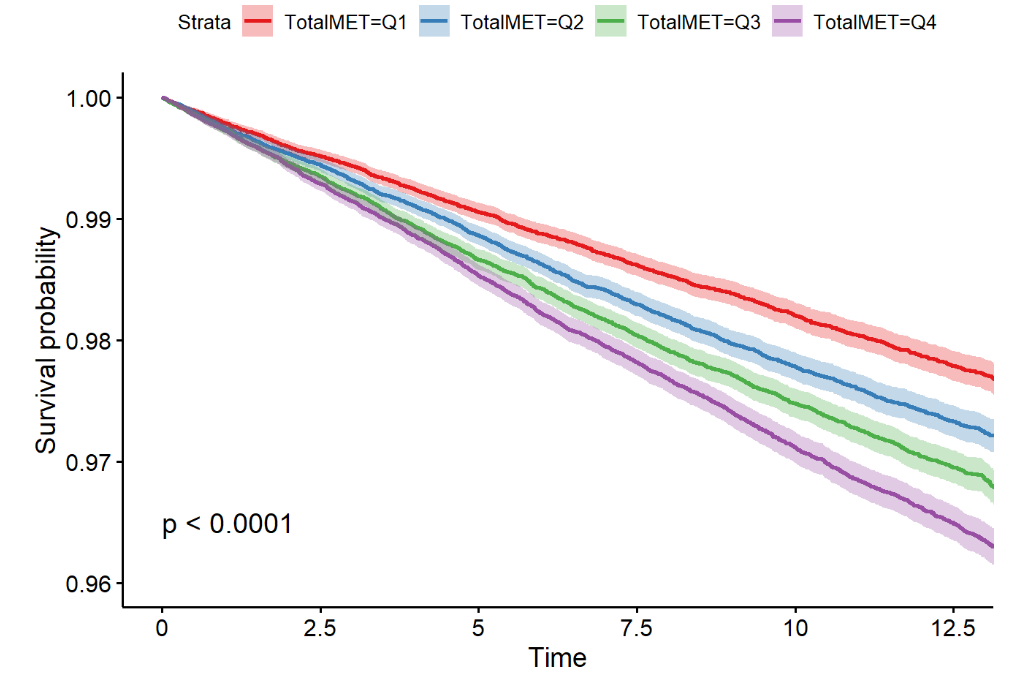


**Supplementary Fig. 8.** Hazard models of predictors of inguinal hernia repair (Women only)


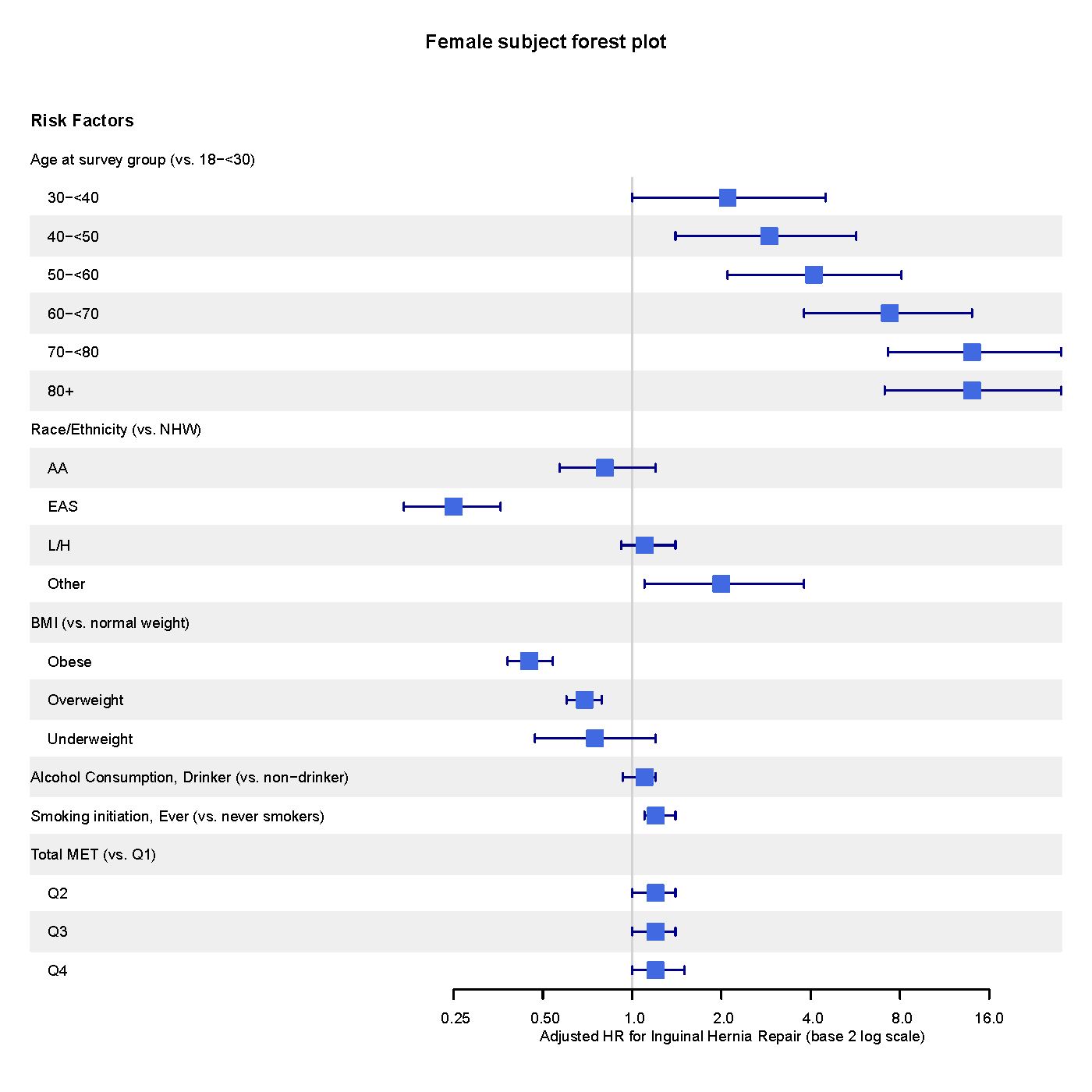


**Supplementary Fig. 9.** Hazard models of predictors of inguinal hernia repair (Men only)


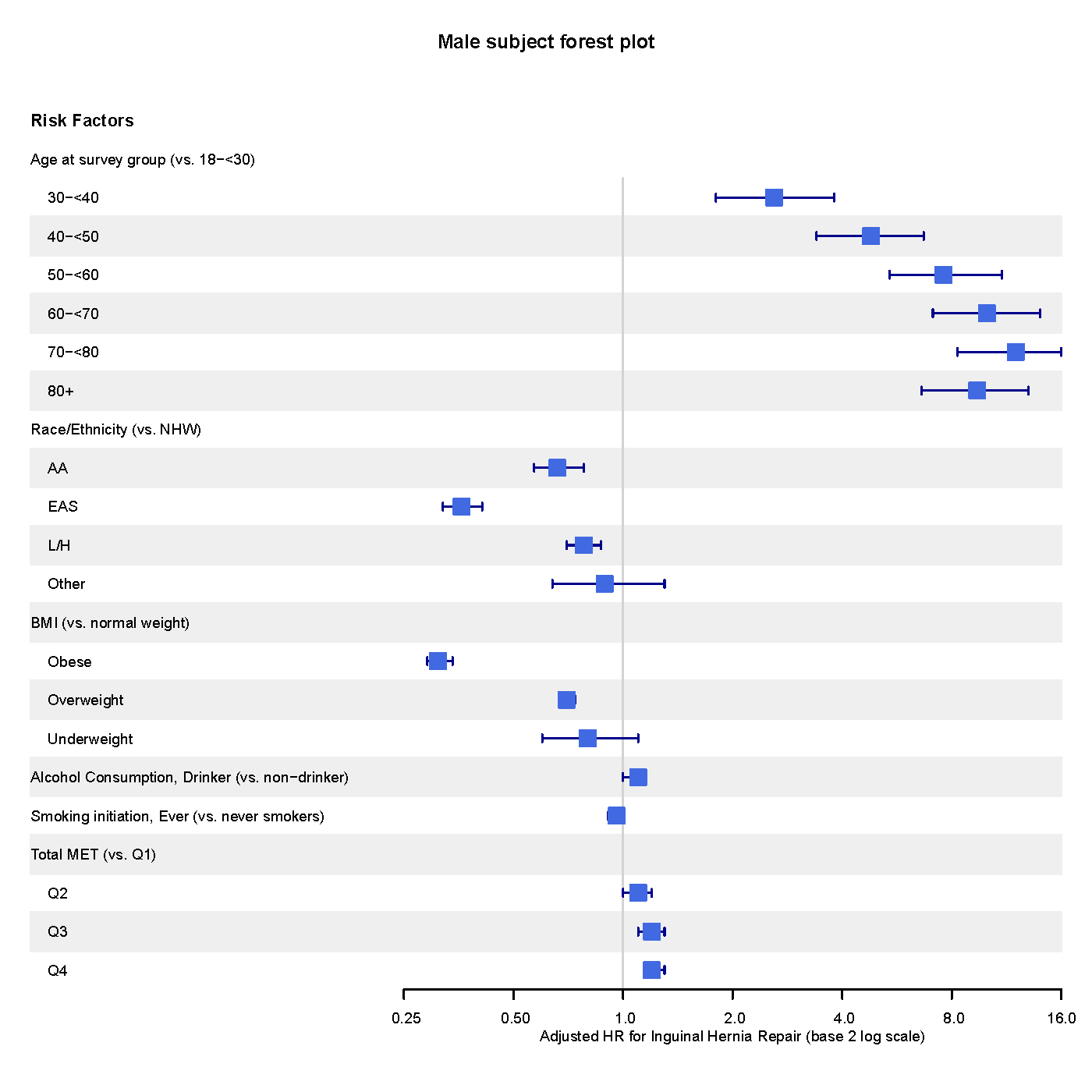

Supplement: Supplementary file 1 — Supplementary file1 (DOCX 1147 KB) [file 10029_2023_2913_MOESM1_ESM.docx]
